# Infective endocarditis of the tricuspid valve

**DOI:** 10.5935/0103-507X.20180026

**Published:** 2018

**Authors:** Sónia Chan, Catarina Faria, Filipa Alçada

**Affiliations:** Serviço de Medicina 1, Centro Hospitalar de Leiria - Leiria, Portugal.; Serviço de Medicina 2, Centro Hospitalar de Leiria - Leiria, Portugal.

**To the Editor**

Infectious endocarditis of the tricuspid valve is rare^(^^1^^,^^2^^)^ and is usually associated with the use of injectable drugs and the manipulation of intravenous devices.^(^^1^^-^^3^^)^

The authors reported the case of a 37-year-old man with drug addiction and hepatitis C, presenting with acute fever, dyspnea, and hemoptysis. At hospital admission, the patient was confused, panting, feverish, tachycardic, and hypotensive. The respiratory murmur was diminished, and he had crackles in the left lung base on auscultation.

**Figure 1:**
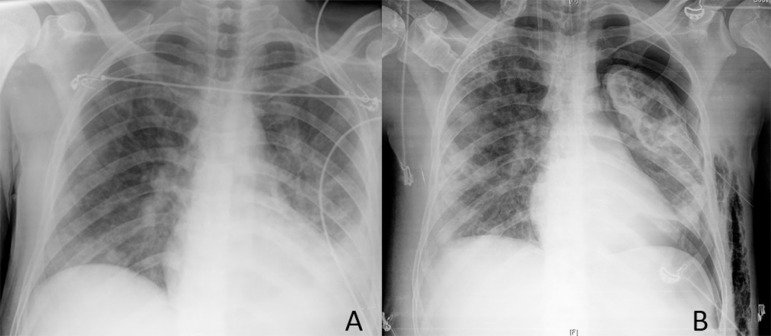
Chest X-ray.

Laboratory analysis revealed leukocytosis with neutrophilia, elevated C-reactive protein, thrombocytopenia, renal damage, metabolic acidosis with acidemia, hyperkalemia, and hyperlactatemia. The patient had bilateral pulmonary condensations and left pleural effusion on chest X-ray (Figure 1A).

**Figure 2:**
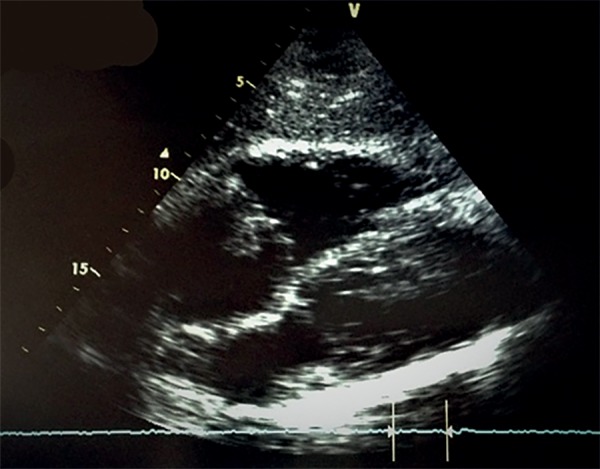
Transthoracic echocardiogram.

The patient was admitted to the intensive care unit for toxic shock with multiple organ dysfunction. During hospitalization, an echocardiogram was performed, which revealed a mobile vegetation 20mm in diameter in the tricuspid valve (Figure 2). Subsequently, methicillin*-*sensitive *Staphylococcus aureus* was isolated from blood cultures.

**Figure 3:**
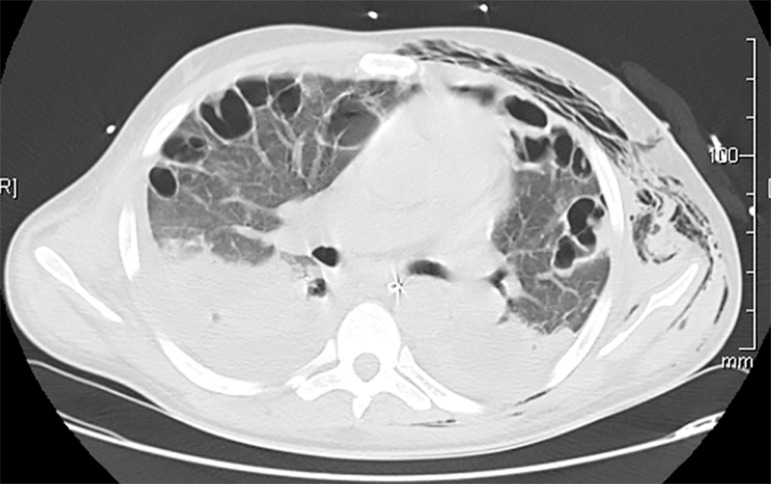
Chest computerized tomography.

Despite the instituted targeted antibiotic therapy, the patient progressed unfavorably. In addition to inotropic and ventilatory support, renal replacement therapy was required. Pulmonary condensations evolved into multiple cavitated lesions, recurrent pneumothorax, and extensive subcutaneous emphysema (Figures 1B and 3).

*S. aureus* is the most common agent in infectious endocarditis associated with injection drug use.^(^^1^^-^^3^^)^ Fever, pulmonary embolism, and bacteremia are signs of right infective endocarditis.^(^^1^^-^^3^^)^ Pulmonary events are present in 80% of cases,^(^^1^^-^^3^^)^ and anemia and hematuria may also be present.^(^^1^^)^ Heart murmur appears only at an advanced stage of the disease.^(^^1^^)^

*Sónia Chan* *Medical Unit 1, Centro Hospitalar de Leiria - Leiria, Portugal.* *Catarina Faria* *Medical Unit 2, Centro Hospitalar de Leiria - Leiria, Portugal.* *Filipa Alçada* *Medical Unit 2, Centro Hospitalar de Leiria - Leiria, Portugal.*
